# The prevalence of tic disorders for children in China: A systematic review and meta-analysis

**DOI:** 10.1097/MD.0000000000004354

**Published:** 2016-07-29

**Authors:** Chunsong Yang, Lingli Zhang, Ping Zhu, Cairong Zhu, Qin Guo

**Affiliations:** a Department of Pharmacy, Evidence-Based Pharmacy Center, West China Second Hospital, Key Laboratory of Birth Defects and Related Diseases of Women and Children; b West China School of Public Health; c Department of Pediatrics, West China Second Hospital, Key Laboratory of Birth Defects and Related Diseases of Women and Children, Sichuan University, Chengdu, Sichuan Province, China.

**Keywords:** children, China, meta-analysis, prevalence, tic disorders

## Abstract

**Background:**

Tic disorders (TD) are common neuropsychiatric disorders among children and adolescents. Still, there is great uncertainty regarding their epidemiology in China. We aim to depict the prevalence of TD for children in China and explore the influence of sex, age, geographic distribution, and diagnostic criteria on the prevalence rates.

**Methods:**

We searched PubMed, EMBASE, four Chinese electronic databases, and relevant lists. Two reviewers independently selected trials, assessed trial quality, and extracted the data.

**Results:**

We included 13 studies investigating 269,571 participants. The sample size ranged from 563 to 216,005 participants. The age of participants ranged from 3 to 16 years. The meta-analysis of the prevalence of TD was 6.1% [95% CI: 0.036–0.100, *I*^2^ = 49.7%]. The prevalence of transient tic disorder (TTD), chronic tic disorder (CTD), and Tourette syndrome (TS) was 1.7% [95% CI = 0.009–0.031, *I*^2^ = 49%], 1.2% [95% CI = 0.007–0.022, *I*^2^ = 48.3%], and 0.3% [95% CI = 0.001–0.008, *I*^2^ = 49.5%], respectively. The prevalence of TD in boys [5.1%, 95% CI = 0.026–0.098, *I*^2^ = 49.3%] was higher than that in girls [2.4%, 95% CI = 0.009–0.065, *I*^2^ = 49.4%]. The prevalence of TD in urban area [2.6%, 95% CI = 0.019–0.034, *I*^2^ = 35.5%] was higher than that in rural area [2.2%, 95% CI = 0.016–0.030, *I*^2^ = 33.9%]. The prevalence of TD in central China [10.7%, 95% CI = 0.043–0.242, *I*^2^ = 49.2%] was higher than that in North China [7.8%, 95% CI = 0.007–0.522, *I*^2^ = 49.9%] and East China [4.4%, 95% CI = 0.015–0.120, *I*^2^ = 49.8%].

**Conclusion:**

TD is a common disease in China, with prevalence differing based on sex, age, and region.

## 1 Introduction

Tic disorders (TD), including transient tic disorder (TTD), chronic motor or vocal tic disorder (CTD), and Tourette syndrome (TS), are common neuropsychiatric disorders among children and adolescents. They are characterized by the presence of repetitive, involuntary, nonrhythmic, sudden movements, or vocalizations that can involve discrete muscle groups.^[1,2]^ Patients with TD may experience subjective discomfort (e.g., pain or injury), sustained social problems (e.g., social isolation or bullying), and emotional problems (e.g., reactive depressive symptoms).^[3]^ These problems may also affect health-related quality of life.^[4]^

A meta-analysis of the worldwide prevalence of TDs indicated that TTD was the most common, with a prevalence of 2.99% (95% confidence interval [CI] = 1.60–5.61), the prevalence of TS was 0.77% (95% CI = 0.39–1.51), and the prevalence of CTD was 1.61% (95% CI = 0.92–2.83).^[5]^ However, this meta-analysis only included two studies conducted in China, and might have missed important TD epidemiology studies published in China because of language limitations. For example, a cross-sectional study^[6]^ conducted with 1752 children in China found that the prevalence of TD was 1.70% in children aged 4 to 6 years, and the prevalence of TTD, CTD, and TS was 0.94%, 0.57%, and 0.19%, respectively. Han^[7]^ surveyed 6000 school children and found that the prevalence of TD was 6.23%, TTD was 3.73%, CTD was 1.6%, and TS was 0.9%.

Given the large population in mainland China, there may be a significant TD disease burden. However, epidemiological data on the prevalence of these disorders are lacking and the prevalence of TDs varies in different studies. Therefore, we firstly present a pooled prevalence of TDs for children by conducting a systematic review of the literature published in China. Secondly, we also explore the prevalence with different characters such as sex age, geographic distribution, and diagnostic criteria in the subgroup analysis.

## 2 Methods

### 2.1 Search strategy

We searched PubMed (1966–March 2015), EMBASE (1974–2014, Issue 3), the Chinese Biomedical Literature Database (CBM, 1978–March 2015), the China National Knowledge Infrastructure (CNKI, 1980–March 2015), the Chinese Science and Technique Journals Database (VIP, 1989–March 2015), and Wanfang Data (http://www.wanfangdata.com/) (1990–March 2015). The bibliographies of relevant articles were screened. Search terms included “incidence,” “prevalence,” “epidemiology,” “Tourette syndrome,” “Tourette disorders,” “tic disorders,” and “tics.” The search was restricted to human studies. Inclusion criteria were as follows: population-based studies conducted in China; participants with age <18 years; the definitions of TD used were drawn from: the *Diagnostic and Statistical Manual of Mental Disorders-III* (DSM-III), DSM-IV, or DSM-IV-Text Revision,^[8–10]^ the *International Classification of Diseases-10* (ICD-10),^[11]^ or the *Chinese Classification and Diagnostic Criteria of Mental Disorders* (CCMD)^[12]^; and data collection methods were questionnaires or face-to-face interviews. The exclusion criteria for studies were s follows: they were reviews or the data for children could not be obtained.

Approval by ethics committee or written consent was not required for the extraction of data on studies already conducted for the purposes of this meta-analysis.

### 2.2 Selection of studies and data extraction

Two review authors independently screened the titles and abstracts of every record identified in the search. Full articles were obtained when the information given in the title or abstract conformed to the selection criteria. Two review authors independently performed data extraction using a standard form that included: time of study, sources of study population, sample size, age of participants, diagnostic criteria used for the disorders, and prevalence of all types of conditions.

### 2.3 Quality assessment

Two review authors independently evaluated the methodological quality of the selected studies using an assessment tool.^[13,14]^ Each study was given a score of 0–8, based on the quality criteria. A score of 7–8 was considered high quality, 4–6 moderate quality, and a score of 0–3 low quality. Disagreements were discussed by the present authors until a consensus was reached.

### 2.4 Statistical methods

The prevalence of TD in the selected studies were combined and reported as proportion with 95% CI. We conducted subgroup analyses based on sex, age distribution, geographic distribution, and diagnostic criteria. We conducted the meta-analysis using meta-analyst software.^[15]^ The *Q* test was used to test the heterogeneity, if *P* value <0.1, the heterogeneity existed. Regardless of the size of heterogeneity, a random effects model was used for statistical analysis.

## 3 Results

### 3.1 Literature search

**Figure 1:**
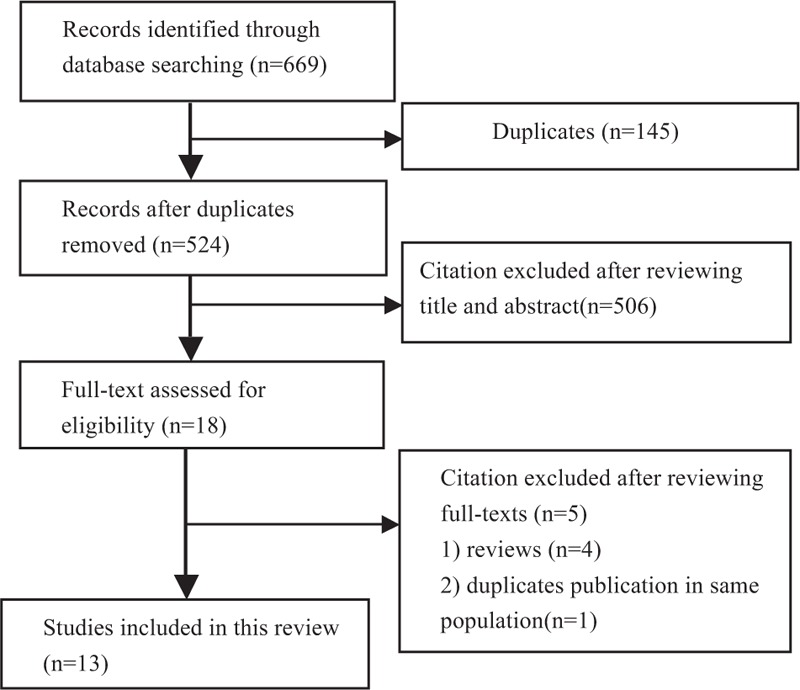
Flow chart of literature screening and selection process.

In total, 669 articles were identified from the electronic searches and screening of reference lists. After removing duplicate articles, and screening titles or abstracts and the full text of the articles, 13 studies met the inclusion criteria and were included in the present review (Fig. 1).

### 3.2 Characteristics of the included studies

The 13 studies included 269,571 participants in total (Table 1). Two studies were published in English and the remaining 11 were in Chinese. The age of participants ranged from 3 to 16 years. The sample size ranged from 563 to 216,005 participants. All studies were single-center studies and all surveys were conducted in school, with the exception of one study conducted in the general population. All studies were conducted during the period from 1983 to 2010. Five studies were conducted in East China, 2 in North China, 2 in Central China, 2 in South China, and 2 in Northeast China.

**Table 1:**
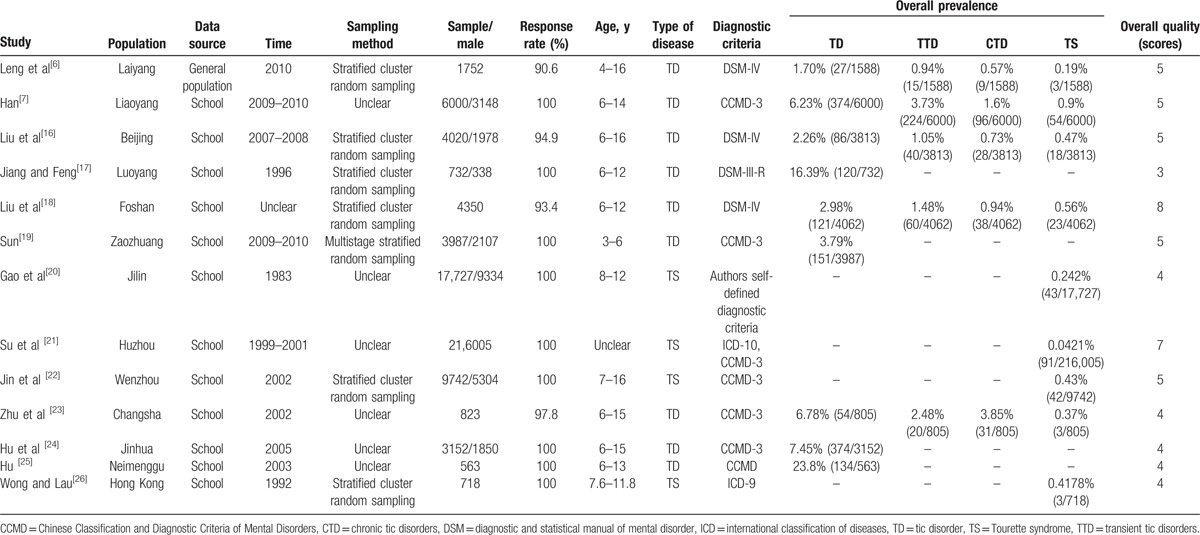
The general characteristic of included study.

For diagnoses, 6 studies used the CCMD, 4 studies used the DSM, 1 study used the ICD-9, 1 study used both the ICD and the CCMD, and 1 study used the authors’ self-defined diagnostic criteria. Nine studies reported the prevalence of TD and TS, and 5 studies reported the prevalence of TTD and CTD.

### 3.3 Quality assessment

The quality scores of the included studies ranged from 3 to 8 points (median 5 points). Two studies^[13,16]^ were rated as good quality, with both studies mostly fulfilling the quality assessment criteria, although 1 study did not estimate the prevalence with 95% CI and in detail by the subgroup. Ten studies were of medium quality, and 1 study^[12]^ was of low quality (scoring 3). All studies defined the target population. In terms of sampling method, 6 studies used stratified and cluster random sampling, 1 used multistage stratified random sampling, and the remaining studies were unclear. In addition, 11 of the included studies reported response rates, which ranged from 90.6% to 100%. However, 7 studies did not clearly describe nonresponders. In 6 studies, the sample was representative of the target population. Whether or not the data collection methods were standardized was unclear in 8 studies. Around 11 studies used validated criteria and 12 did not estimate the prevalence with 95% CI and in detail by subgroup. The quality scores of the included studies are shown in Table 2.

**Table 2:**
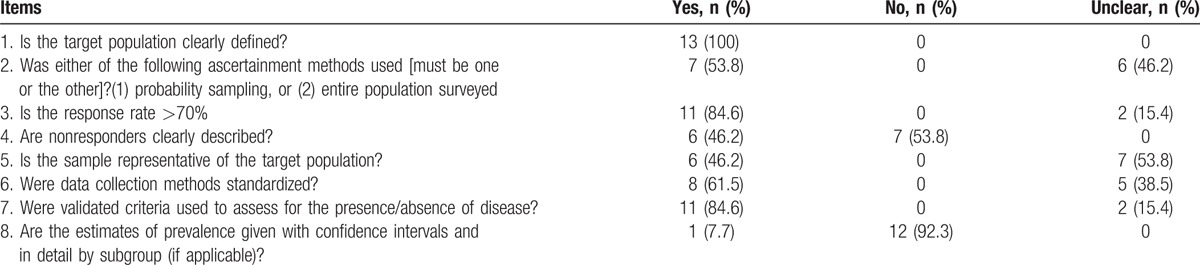
The quality assessment of included studies.

### 3.4 Prevalence

**Figure 2:**
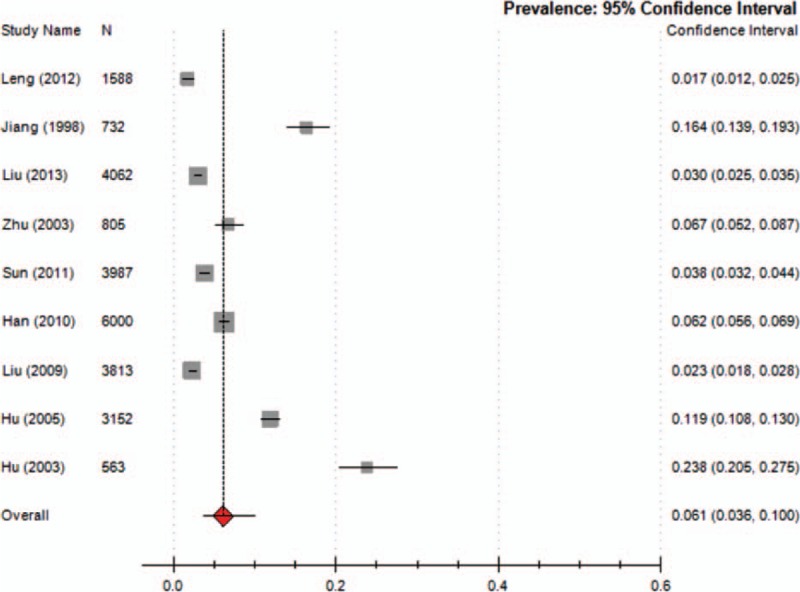
Meta-analysis of the prevalence of TD in children. TD = tic disorder.

**Figure 3:**
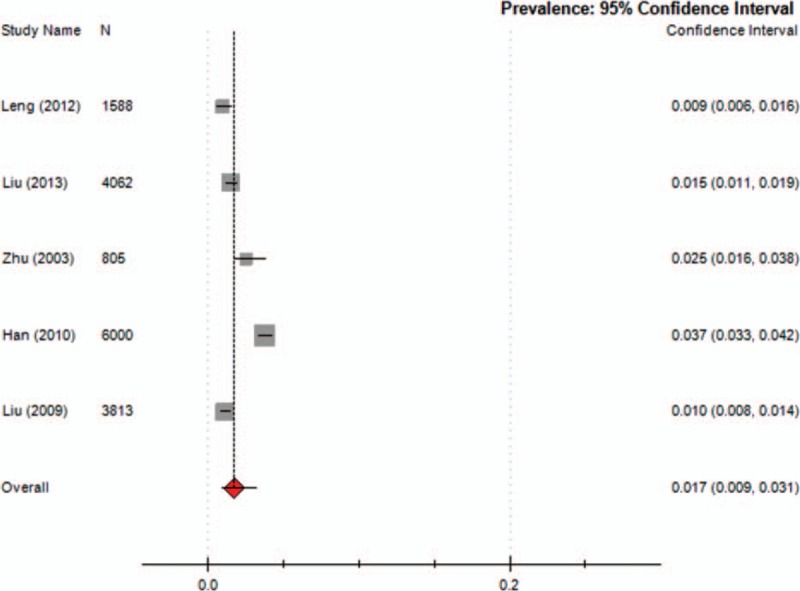
Meta-analysis of the prevalence of TTD in children. TTD = transient tic disorders.

**Figure 4:**
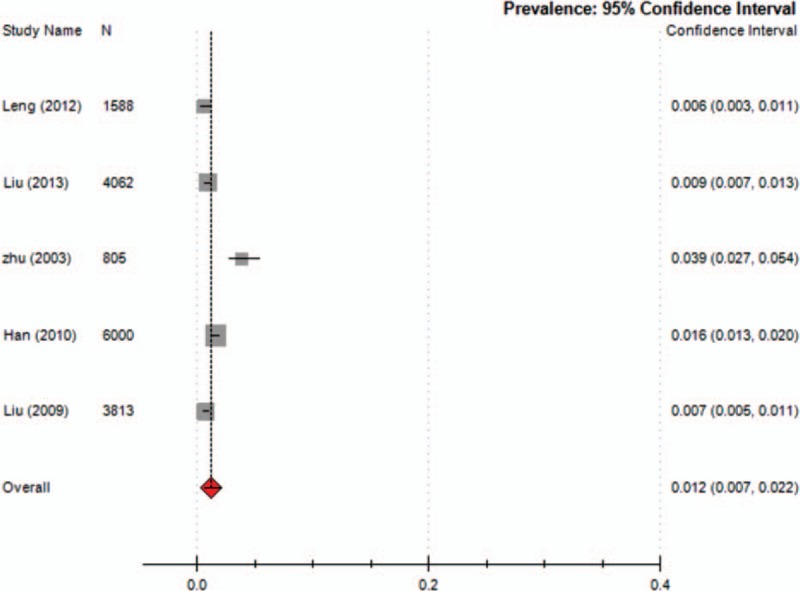
Meta-analysis of the prevalence of CTD in children. CTD = chronic tic disorders.

**Figure 5:**
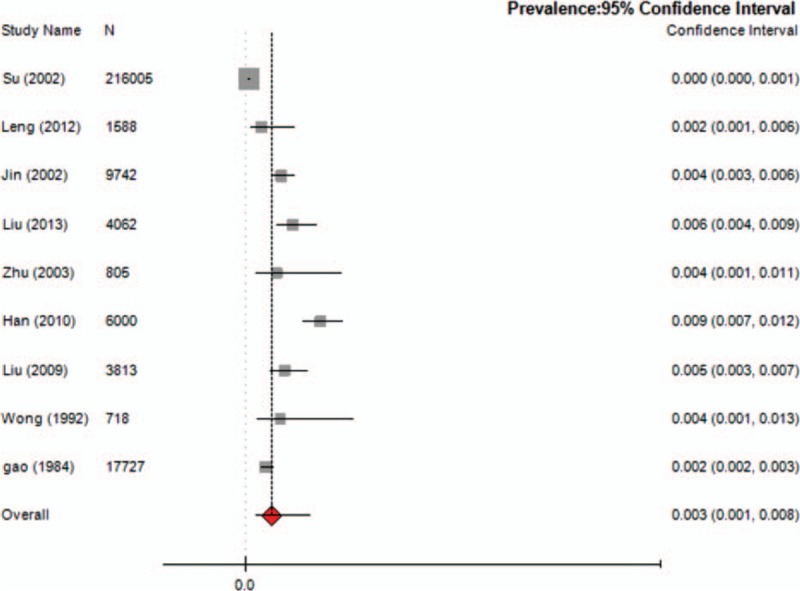
Meta-analysis of the prevalence of TS in children. TS = Tourette syndrome.

Nine studies reported the prevalence of TD in children, which ranged from 1.7% to 23.8% (median 6.23%), with a combined prevalence of 6.1% (95% CI = 0.036–0.100; *I*^2^ = 49.7%) (Fig. 2). Five studies reported the prevalence of TTD and CTD. The prevalence of TTD ranged from 0.94% to 3.73% (median 1.48%) and that of CTD ranged from 0.57% to 3.85% (median 0.94%). The meta-analysis of the overall prevalence for TTD was 1.7% (95% CI = 0.009–0.031; *I*^2^ = 49%; *Q* = 0.990; *P* = 0.000) (Fig. 3) and for CTD was 1.2% (95% CI = 0.007–0.022; *I*^2^ = 48.3%; *Q* = 0.983; *P* = 0.000) (Fig. 4). Nine studies reported the prevalence of TS, ranging from 0.0421% to 0.9% (median 0.42%) with a combined prevalence of 0.3% (95% CI = 0.001–0.008; *I*^2^ = 49.5%; *Q* = 0.998; *P* = 0.000) (Fig. 5).

### 3.5 Subgroup analysis

#### 3.5.1 Prevalence based on sex

Five studies provided data on the prevalence of TD for boys and girls, reporting ratios ranging from 1.42 to 3.26 for TD. Three studies^[6,16,18]^ reported the prevalence of TTD and CTD, and 5 studies reported the prevalence of TS. These studies reported boy:girl ratio ranges of 2.22 to 3.68 for TTD, 1.57 to 2.79 for CTD, and 2.17 to 10.6 for TS. The subgroup meta-analysis for sex-based prevalence is presented in Table 3.

**Table 3:**
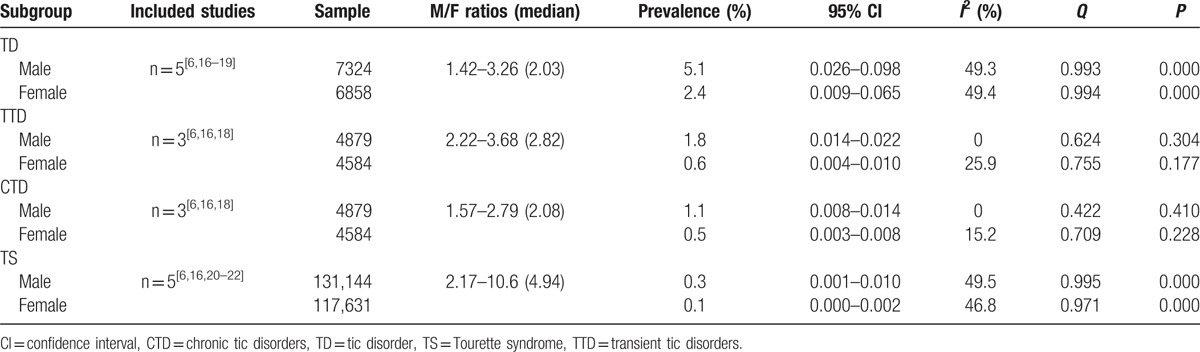
The meta-analysis for subgroup of gender prevalence.

#### 3.5.2 Prevalence based on age distribution

Five studies reported the prevalence of TD and TS in different age groups. Five studies provided data on the relationship between age and prevalence. The subgroup meta-analysis for age distribution-based prevalence is presented in Table 4.

**Table 4:**
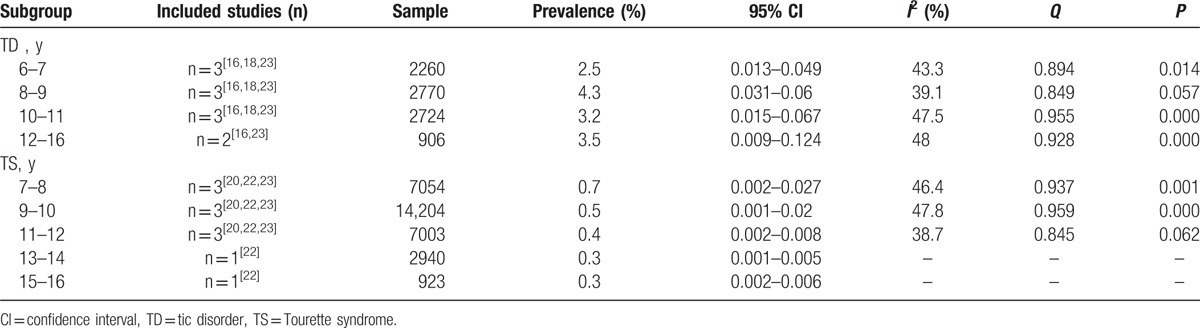
The meta-analysis for subgroup of age distribution.

#### 3.5.3 Prevalence based on geographic distribution

Five studies explored the association between geographic distribution and the prevalence of TD. The prevalence of TD in central China (10.7%) was higher than that in North China (7.8%) and East China (4.4%). The prevalence of TS in Northeast China (0.5%) and South China (0.5%) was higher than that in East China (0.1%). The subgroup meta-analysis for geographic distribution-based prevalence is presented in Table 5.

**Table 5:**
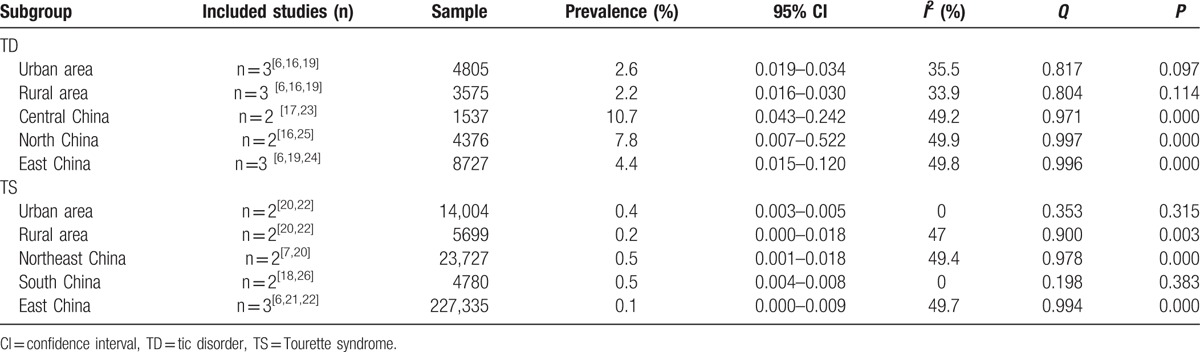
The meta-analysis for subgroup of geographic distribution.

#### 3.5.4 Prevalence based on diagnostic criteria

Six studies used the CCMD diagnostic criteria, 4 studies used the DSM,^[6,16,17,18]^ 1 study^[21]^ used both the ICD-10 and the CCMD-3, 1 study^[26]^ used the ICD-9, and 1 study^[20]^ used the authors’ self-defined diagnostic criteria. With the CCMD diagnostic criteria, the prevalence of TD ranged from 3.79% to 23.8%. With the DSM diagnostic criteria, the prevalence of TD ranged from 1.7% to 16.39%. The subgroup meta-analysis for diagnostic criteria-based prevalence is presented in Table 6.

**Table 6:**
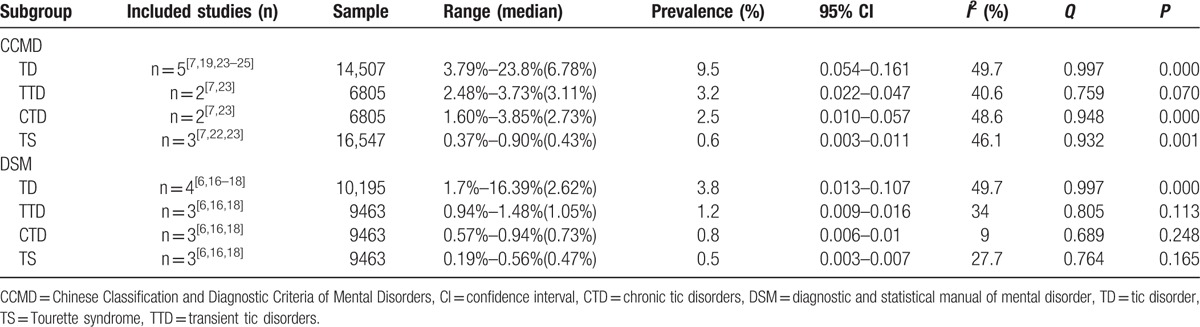
The meta-analysis for subgroup of diagnostic criteria.

## 4 Discussion

The present systematic review identified relevant studies and provided general information on the prevalence of TD in children in China. We found an overall prevalence of ∼6%. Our meta-analysis also showed that TTD (1.7%) was the most common type of TD in children, followed by CTD (1.2%) and TS (0.3%). The prevalence of TTD, CTD, and TS in China was lower than the reported worldwide rates (TTD 2.99%, CTD 1.61%, and TS 0.77%).^[5]^ However, the overall prevalence of TD (6.1%) in China was relatively high. Possible reasons for this are as follows: 9 of the included studies reported the prevalence of TD, but only 5 reported the prevalence of TTD and CTD meaning that data on the prevalence of TTD and CTD was limited; our study included 13 studies with participants ranging in age from 3 to 16 years. However, the meta-analysis of the worldwide prevalence of TD included 26 studies with participants aged from 0 to 18 years, with most focusing on patients aged 6–15 years,^[5]^ age may have influenced the prevalence rates obtained. The differences in data source might also result in differences in the data. In our study, almost all data were derived from school samples, whereas data collected in the study by Knight et al^[5]^ were from a variety of sources (door-to-door surveys, mailed surveys, telephone surveys, and school surveys).

The study by Knight et al^[5]^ found that the prevalence of TD was higher in all studies performed in special education populations, but this study did not discuss the reason. In our study, there was no such study that presented the data of children with special education, so we did not conclude whether the special education influence the prevalence of TD.

To explore the influence of specific factors (sex, geographic distribution, and diagnostic criteria) on prevalence, we conducted subgroup analyses. Our results showed that TD and its various subtypes were more common among boys than girls and preferentially affected boys, which was consistent with the international consensus. The prevalence of TD in younger age groups appeared to be higher than that in older age groups, with TD mainly concentrated in those aged 8 to 9 years, and TS in those aged 7 to 8 years. In our study, we did not find significant differences in the prevalence of TD and TS between age groups. This may be explained by the fact that some of the studies in our review only reported the general prevalence of TDs, and did not report the prevalence by disease type, sex, age, and geographic distribution, making it difficult to conduct a meta-analysis of the prevalence based on different subgroups. In addition, the different diagnostic criteria used in the included studies might have influenced prevalence. We found that the prevalence of TD in urban areas was higher than that in rural areas; some studies found that environmental pollution may result in TD, and children in urban areas may therefore be more exposed to environmental pollution.

The prevalence of TD using the CCMD diagnostic criteria was higher than that using the DSM diagnostic criteria, which was consistent with the study by Scharf et al^[27]^ which concluded that diagnostic criteria might influence the prevalence of TS. In the CCMD diagnostic criteria, CTD and TS diagnosis specifies no remission period of >2 months in a 1-year period; however, the DSM diagnostic criteria specifies the course of the disease as >1 year and an intermittent time of no >3 months. Therefore, the prevalence of TD using the CCMD diagnostic criteria may be higher than that using the DSM diagnostic criteria. Future epidemiological studies should try to use unified disease diagnostic criteria.

In general, the quality of the included studies was not high: 46.2% of the studies did not clearly describe the sampling methods, different sampling methods may result in differences in prevalence and it was unclear whether the samples were representative of the target population. In 61.5% of the studies, it was not clear if the data collection methods were standardized, which might introduce bias into data collection, and 92.3% of the studies did not give estimates of prevalence with confidence intervals, making it difficult to judge data variation. All of these factors might have influenced our approach to data analysis and interpretation of the results, and future studies should provide more methodological detail.

Our study had some limitations. First, most studies were located in the north and south of China, with data for the west of China lacking, meaning geographical distribution was unbalanced and the sample was not sufficiently representative. Therefore, a large-scale multicenter study on the epidemiology of TD across different areas in China is required. Second, we found moderate heterogeneity in our meta-analyses of prevalence of all types of TD, which may be attributed to the differences in sample size and the included studies using different diagnostic criteria. Third, the literature search was limited to articles published in Chinese or English. Fourthly, some of the included studies only reported the general prevalence of TD, and did not report the prevalence by disease type, sex, age, and geographic distribution, making it difficult to conduct meta-analyses of prevalence based on different subgroups. Finally, because of the limited included studies, this study could not consider the interaction effect between different factors. Future research should focus on overcoming this limitation.

In conclusion, TD is a common disease in China, with prevalence differing based on sex, age, and region.
